# The Impact of Disease Severity and Symptoms on Anxiety and Depression in Individuals With Idiopathic Pulmonary Fibrosis

**DOI:** 10.1016/j.chest.2026.03.016

**Published:** 2026-03-30

**Authors:** Zhe Wu, Karen Moor, Marlies S. Wijsenbeek, Christina Schlecker, Gerrit Weimann, Toby M. Maher, Philip L. Molyneaux

**Affiliations:** a National Heart and Lung Institute, Imperial College London, London, England; b Department of Respiratory Medicine, Erasmus Medical Center, Rotterdam, The Netherlands; c Boehringer Ingelheim International GmbH, Ingelheim am Rhein, Germany; d Division of Pulmonary, Critical Care and Sleep Medicine, University of Southern California, Los Angeles, CA

**Keywords:** anxiety, cough, depression, dyspnea, fatigue, idiopathic pulmonary fibrosis

## Abstract

**Background:**

Idiopathic pulmonary fibrosis (IPF) is a debilitating, life-limiting fibrotic lung disease. Symptoms including breathlessness, cough, and fatigue, together with disease severity, contribute to psychological burden, yet their relationship with anxiety and depression remains unclear.

**Research Question:**

In patients with IPF, what is the prevalence and severity of anxiety and depression, and what are their associations with symptoms and disease severity?

**Study Design and Methods:**

This observational study prospectively recruited patients with incident IPF. Hospital Anxiety and Depression Scale (HADS), Dyspnea-12, Leicester Cough Questionnaires, cough visual analog scales, Living with IPF (L-IPF), and lung function were assessed at baseline and 12 months.

**Results:**

Two hundred sixty-nine patients with IPF (mean age, 73.5 years; 81% male) were recruited; 157 completed follow-up. Mean FVC % predicted ± SD was 79% ± 14.7%. At baseline, the prevalence of anxiety and depression (HADS score ≥ 8) was 27.5% and 28.3%, respectively (suggestive HADS 8-10: 18.6% and 14.9%; probable HADS ≥ 11: 8.9% and 13.3%, respectively). On multivariable analysis, worse Dyspnea-12 and poorer L-IPF energy were independent factors for both anxiety and depression at baseline; younger age was a factor for anxiety. Baseline lung function did not correlate with HADS anxiety or HADS depression. At follow-up, HADS anxiety scores increased numerically, below reported minimal clinically important difference values, with no change in HADS depression scores. Younger age, higher Dyspnea-12 scores, and poorer L-IPF energy were associated with greater increases in anxiety. Moderate or weak correlations were observed between changes in HADS and longitudinal changes in patient-reported outcomes (worsening breathlessness, cough, and energy levels). Changes in FVC % predicted did not correlate with HADS.

**Interpretation:**

Our results show that anxiety and depression are common in patients with IPF and are associated with more dyspnea and lower energy. The mental health burden experienced by patients with IPF needs better recognition and might be helped by targeted symptom management.

**Take-Home Points**

**Research Question:** In patients with idiopathic pulmonary fibrosis, what is the prevalence and severity of anxiety and depression, and what are their associations with symptoms and disease severity?

**Results:** Anxiety and depression were prevalent (27.5% and 28.3%, respectively) and significantly associated with greater breathlessness (Dyspnea-12) and reduced energy levels, whereas baseline lung function did not correlate with Hospital Anxiety and Depression Scale scores.

**Interpretation:** Our results show that psychological distress in idiopathic pulmonary fibrosis is more closely linked to perceived symptom burden rather than objective lung function, highlighting the need for targeted symptom management and psychological support in holistic patient care.

In patients with interstitial lung disease (ILD), including idiopathic pulmonary fibrosis (IPF), the prevalence of anxiety and depression is high, and is associated with worse outcomes.^1^, ^2^, ^3^, ^4^, ^5^ Estimates for the prevalence of anxiety and depression in people with ILD range from 21% to 60% for anxiety and 14% to 49% for depression.^1^, ^2^, ^3^, ^4^, ^5^ There are relatively few studies that have reported on the prevalence of anxiety and depression in IPF specifically.

Anxiety and depression significantly influence quality of life (QoL) in people with IPF.^1^ Left untreated, anxiety and depression are associated with other adverse outcomes including poor compliance with treatment, early dropout from rehabilitation, increased dyspnea, social isolation, increased health care utilization, and premature mortality.^2^ The cause of anxiety and depression in people with IPF is complex and likely involves a combination of different external, personal, and disease-related factors that vary between individuals.^2^

People with IPF experience a high disease and symptom burden, and the commonly reported symptoms of IPF, such as cough, dyspnea, and impaired physical ability, are associated with a negative psychological burden.^2^ Most patients with IPF present with breathlessness, which becomes progressively worse, and patient-reported dyspnea has been shown to be a strong predictor of mortality in patients with IPF.^6^^,^^7^

The association between IPF symptoms and disease severity (in terms of impaired lung function) with anxiety and depression remains underexplored. Severe cough has been associated with worse QoL in patients with IPF,^8^ while worse cough-related QoL has in turn been associated with more severe physiological impairment.^9^ Chronic cough and fatigue, prevalent in approximately 80% and up to 95% of patients with IPF, respectively, have been found to be associated with anxiety and depression.^10^, ^11^, ^12^ While anxiety is an emotional response to breathlessness, it can also increase the perception of breathlessness.^7^^,^^13^ Dyspnea has been shown to correlate with anxiety and depression in patients with ILD and IPF.^5^^,^^14^^,^^15^ An Australian IPF registry study found that dyspnea and cough contributed to prolonged anxiety and depression, but this was not related to lung function decline.^5^ In contrast, a small prospective ILD cohort study (including patients with IPF) found that FVC was an independent predictor of depressive symptoms at baseline.^15^ In a large UK prospective cohort study of a general population, impaired lung function was associated with a higher risk of developing depression.^16^

Overall, there is a lack of comprehensive evaluation of the relationship between IPF symptoms, disease severity, and psychological distress, particularly the impact of lung function decline on the development of anxiety and depression. The objectives of this study were to report the prevalence and severity of anxiety and depression in a population of patients with IPF and determine its association with respiratory symptoms, disease severity, and how these relationships change over time.

## Study Design and Methods

### Study Design and Participants

This analysis was conducted using data from the Progressive Fibrosing Lung Disease (PROFOUND) study, an observational study conducted at a tertiary ILD center (Royal Brompton Hospital, London, England). PROFOUND prospectively recruited patients with an incident diagnosis of IPF according to the American Thoracic Society/European Respiratory Society/Japanese Respiratory Society/Latin American Thoracic Association guidelines.^17^^,^^18^

Incident cases of IPF between March 2021 and December 2023 were prospectively recruited with diagnoses established through multidisciplinary team consensus. Eligible patients had a new diagnosis of IPF within the preceding 6 months and were treatment-naïve, having not received antifibrotic therapy. Patients with a history within the preceding 6 weeks of self-reported upper or lower respiratory tract infection, antibiotic use, or acute exacerbation of IPF were excluded. Approval for the study was obtained from the East of England-Essex Research Ethics Committee (Reference 20/EE/0261), and all participants provided written informed consent.

Participants underwent assessments at baseline and 12 months. Demographic data, including age, BMI, and smoking history, were collected at baseline. At each study visit, pulmonary function testing was performed, including measurement of FVC and diffusing capacity of the lungs for carbon monoxide (Dlco). Patient-reported outcomes (PROs) were assessed using validated instruments, including the Hospital Anxiety and Depression Scale (HADS; subscales for anxiety [HADS-A] and depression [HADS-D]; range, 0-21; higher scores depicting worse QoL), Leicester Cough Questionnaire (LCQ) (range, 3-21, with higher scores indicating better QoL), Dyspnea-12 (range, 0-36, with higher scores indicating greater breathlessness severity), a visual analog scale (VAS) (range, 0-100 mm, with higher scores indicating more severe cough) for cough severity, and Living with IPF (L-IPF) (range, 0-100, with higher scores indicating better health status). Concomitant medication use, including angiotensin-converting enzyme inhibitors, long-term oxygen therapy, antidepressants, and proton pump inhibitor use, was documented. Anonymized data were systematically recorded using the REDCap platform. This study is ongoing, and the data presented here are an analysis based on data as of December 2024.

### Statistical Analysis

The prevalence of anxiety and depression was estimated using the results of the HADS-A and HADS-D scores, respectively, at both baseline and 12 months; no disorder was defined as HADS-A/HADS-D score ≤ 7, suggestive disorder as HADS-A/HADS-D score 8-10, and probable disorder as HADS-A/HADS-D score ≥ 11.^19^, ^20^ Clinical variables associated with anxiety (HADS-A) and depression (HADS-D) were assessed at baseline by univariable analysis using the general linear model. Unstandardized coefficients from a regression model and 95% CIs were calculated for the association between different factors and baseline HADS scores. Statistically significant variables (*P* < .05) from the univariable analyses were entered into multivariable linear regression analyses for baseline HADS anxiety and depression scores. Four multivariable linear regression models were tested for baseline HADS-A scores and 4 models were tested for HADS-D scores. To avoid the effects of multicollinearity, cough variables (LCQ and VAS) were analyzed in separate models. Longitudinal changes in HADS score (from baseline to 12 months) were then assessed with a linear mixed-effects model. Time was modeled as a categorical independent fixed effect, and a random intercept for participant ID was included to account for repeated measurements. Covariates identified as clinically and statistically relevant in the baseline HADS regression analyses (age, cough VAS, Dyspnea-12, and L-IPF energy) were incorporated both as time-invariant predictors using baseline values and as time-varying covariates to capture within-person change. To account for multiple comparisons in the linear mixed-effects models, *P* values for fixed effects were adjusted using the Holm-Bonferroni sequential correction method.

To assess the relationship between changes in disease severity (FVC) and symptoms (cough VAS, LCQ, Dyspnea-12, and L-IPF energy) and changes in anxiety and depression over 12 months, Spearman correlation analyses were performed. These correlation analyses were considered exploratory, hypothesis-generating analyses; therefore, no correction for multiple comparisons was applied, and nominal *P* values are reported. Changes in PROs (cough VAS, LCQ, L-IPF energy, HADS-A, and HADS-D) after 12 months were compared for patients defined as stable or progressive. Progression was defined as a relative decline in % predicted FVC ≥ 10% or death within 12 months.

## Results

### Characteristics of the Study Population

Overall, 269 of 313 patients recruited into the study completed the HADS questionnaire; of these, 157 completed follow-up at 12 months at time of analysis. At baseline, the mean age of patients was 73.5 years (Table 1). Patients were predominantly male (81%), with moderately impaired lung function (mean FVC % predicted ± SD, 79% ± 14.7%; Dlco, 49% ± 14%) (Table 1, Table 2). The mean baseline HADS-A and HADS-D scores ± SD were 5.1 ± 3.7 and 5.6 ± 3.9, respectively. At 12-month follow-up, the mean HADS-A score ± SD was 5.5 ± 3.9, whereas the mean HADS-D score ± SD was 5.7 ± 3.5 (Table 2). A total of 187 patients received antifibrotic therapy, and there was no difference in baseline anxiety or depression or change in HADS score over 12 months when compared with patients who did not receive therapy.

**Table 1:** Patient Characteristics at Baseline (N = 269)

**Characteristic**	Value
Age, y	73.5 [7.2]
Male sex	218 (81.0)
BMI, kg/m^2^	28.5 [4.7]
Smoking history	
Former or current	176 (65)
Never	92 (34)
Long-term oxygen therapy	31 (11)
ACEi therapy	58 (21)
Antidepressant therapy	24 (8)
Proton pump inhibitor use	150 (55)

Data are presented as mean [SD] or frequency (%). ACEi = angiotensin-converting enzyme inhibitor.

**Table 2:** Demographic Data at Baseline and 12-Month Follow-Up

Variable	Baseline (n = 269)		Follow-Up (n = 157)	
	Value	Range	Value	Range
Pulmonary function tests				
FEV_1_ % predicted	84 [15.4]	26-129	83 [15.5]	38-123
FVC % predicted	79 [14.7]	39-127	79 [16.0]	33-124
Dlco % predicted	49 [14.0]	20-106	46 [12.8]	13-78
HADS anxiety	5.1 [3.7]	0-18	5.5 [3.9]	0-19
HADS depression	5.6 [3.9]	0-18	5.7 [3.5]	0-15
Dyspnea-12	13 [8.8]	0-36	12 [9.0]	0-36
Cough VAS	32 [24.0]	0-91	30 [23.4]	0-93
LCQ	16 [2.7]	8-21	16 [2.7]	8-21
L-IPF impacts	49 [25.6]	0-100	47 [26.9]	3-100
L-IPF cough	29 [23.3]	0-95	27 [23.2]	0-90
L-IPF dyspnea	30 [18.9]	3-85	30 [20.5]	3-89
L-IPF energy	42 [22.0]	0-91	44 [21.9]	0-91

Data are presented as mean [SD], frequency (%), or otherwise indicated. Dlco = diffusing capacity of the lungs for carbon monoxide; HADS = Hospital Anxiety and Depression Scale; LCQ = Leicester Cough Questionnaire; L-IPF = Living with Idiopathic Pulmonary Fibrosis; VAS = visual analog scale.

### Prevalence of Anxiety and Depression

**Figure 1:**
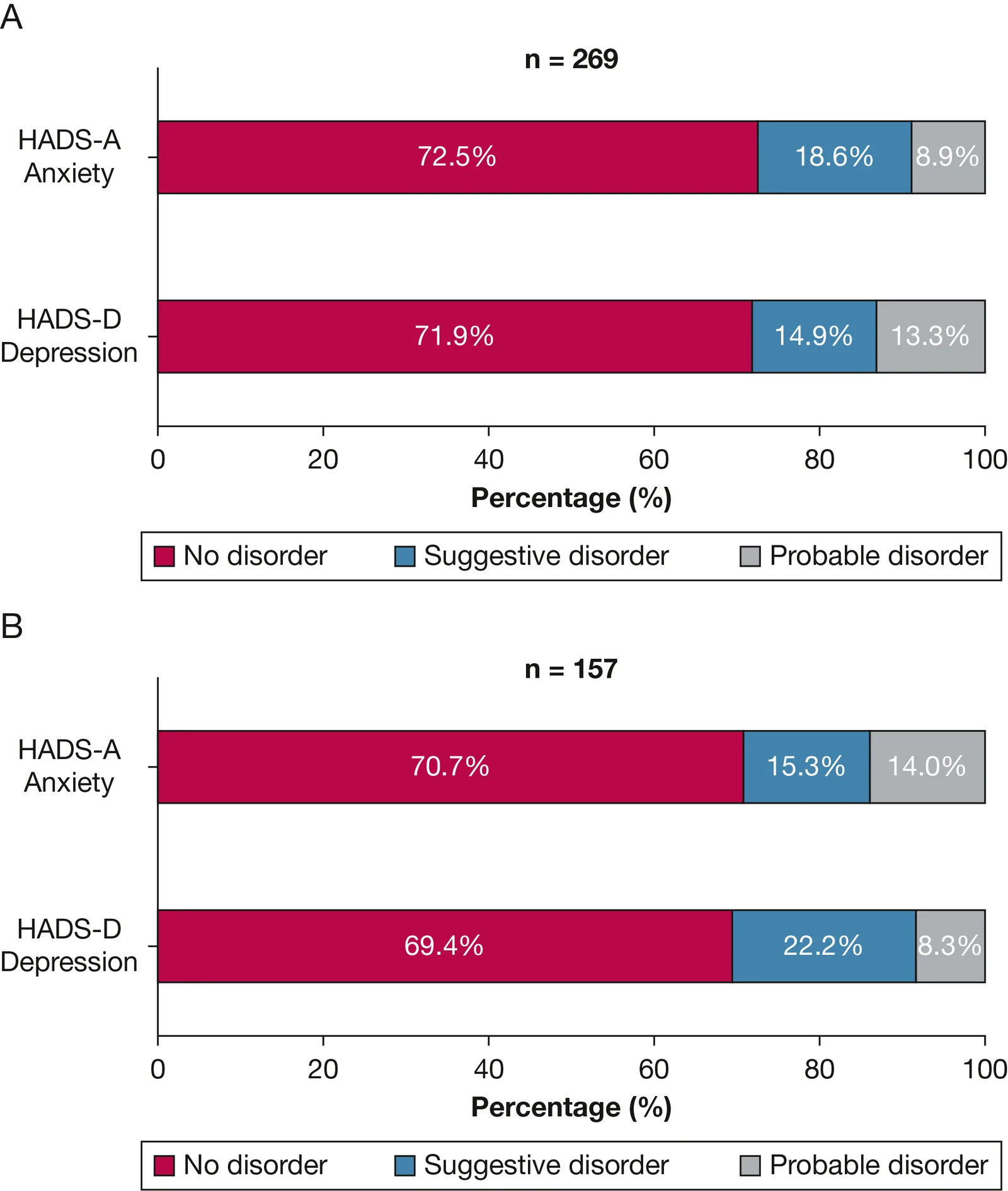
A, B, Prevalence of anxiety (HADS-A) and depression (HADS-D) at (A) baseline (n = 269) and (B) 12 mo (n = 157). HADS-A = Hospital Anxiety and Depression Scale anxiety subscale; HADS-D = Hospital Anxiety and Depression Scale depression subscale; No disorder = HADS-A/HADS-D score ≤ 7; Suggestive disorder = HADS-A/HADS-D score 8-10; Probable disorder = HADS-A/HADS-D score ≥ 11.

The prevalence of anxiety and depression at baseline (HADS score ≥ 8: suggestive and probable disorder) was 27.5% and 28.3%, respectively (Fig 1A). At baseline, 18.6% (50/269) and 8.9% (24/269) of participants exhibited suggestive (HADS score 8-10) and probable (HADS score ≥ 11) levels of anxiety disorder, respectively, while 14.9% (40/269) and 13.3% (36/269) demonstrated suggestive (HADS score 8-10) and probable (HADS score ≥ 11) levels of depressive disorder. At 12 months, the prevalence (HADS score ≥ 8: suggestive and probable disorder) was 29.3% for anxiety and 30.6% for depression (Fig 1B). Overall, 15.3% (24/157) and 14.0% (22/157) of participants showed suggestive (HADS score 8-10) and probable (HADS score ≥ 11) anxiety disorder, respectively, and 22.2% (35/157) and 8.3% (13/157) showed suggestive (HADS score 8-10) and probable (HADS score ≥ 11) depressive disorder. During the follow-up period, 47 patients died. Of those patients, 25.5% had a baseline HADS-A score ≥ 8, whereas 36.0% had a baseline HADS-D score ≥ 8.

### Correlations With Anxiety and Depression: Baseline

On univariable analysis, several clinical variables were significantly associated (*P* < .05) with baseline anxiety and depression. These included Dyspnea-12 scores, cough VAS, LCQ total score, and multiple domains of the L-IPF questionnaire, specifically impacts, cough, dyspnea, and energy subscales, as well as smoking history and use of oxygen therapy. In addition, younger age was significantly associated with anxiety, whereas higher BMI, lower FVC % predicted, and lower Dlco % predicted were significantly associated with depression (e-Table 1).

However, on multivariable modeling, only Dyspnea-12 score, L-IPF energy, and age remained independent factors for worse anxiety score. Dyspnea-12 and L-IPF energy scores remained associated with worse depression scores at baseline (Table 3). After applying multiple-testing correction, the same covariates remained significant, while the effects of time and baseline cough severity were confirmed to be nonsignificant (e-Appendix 1).

**Table 3:** Clinical Variables Associated with Anxiety (HADS-A) and Depression (HADS-D) at Baseline on Multivariable Linear Regression Models

HADS-A				HADS-D			
Variables	β	95% CI (β)	*P* Value	Variables	β	95% CI (β)	*P* Value
Model 4 *R*^2^ = 29.9%			< .001	Model 8 *R*^2^ = 44.4%			< .001
Smoking history	0.036	−2.605 to 2.678	.979	Smoking history	0.082	−2.606 to 2.780	.952
Oxygen therapy	0.000	−1.539 to 1.539	1.000	Oxygen therapy	−0.648	−2.212 to 0.915	.414
Cough VAS	0.002	−0.018 to 0.021	.860	Cough VAS	0.002	−0.016 to 0.021	.819
Dyspnea-12	0.127	0.064 to 0.190	< .001	Dyspnea-12	0.118	0.055 to 0.181	< .001
L-IPF energy	0.044	0.020 to 0.068	< .001	L-IPF energy	0.086	0.063 to 0.109	< .001
Age	−0.124	−0.188 to −0.061	< .001	Dlco % predicted	0.010	−0.026 to 0.046	.581
				FVC % predicted	−0.001	−0.033 to 0.031	.942
				BMI	−0.013	−0.107 to 0.080	.777

Data are presented with unstandardized (β) correlation coefficients with 95% CIs for associations between factors and Hospital Anxiety and Depression Scale scores. Model 4 was used for baseline HADS-A scores and model 8 was used for baseline HADS-D scores. Dlco = diffusing capacity of the lungs for carbon monoxide; HADS-A = Hospital Anxiety and Depression Scale anxiety subscale; HADS-D = Hospital Anxiety and Depression Scale depression subscale; L-IPF = Living with Idiopathic Pulmonary Fibrosis; VAS = visual analog scale.

### Changes in Anxiety and Depression Over Time

From baseline to 12 months, HADS-A score increased by 0.48 points in the unadjusted model (*P* = .044) but was not statistically significant when adjusted for baseline covariates (*P* = .057). A younger age (β = −0.125; *P* = .003), higher Dyspnea-12 scores (β = 0.104; *P* = .011), and poorer L-IPF energy levels (β = 0.044; *P* = .006) at baseline were predictors of increased anxiety over time (e-Table 2). HADS-D scores did not change over time in either the unadjusted or adjusted models. Although the adjusted model highlighted significant associations with baseline Dyspnea-12 (β = 0.156; *P* = .004) and L-IPF energy scores (β = −0.045; *P* = .016), the overall model for depression was not statistically significant (e-Table 2). Therefore, these findings likely reflect stable between-subject differences in anxiety and depression scores, rather than within-subject changes over time.

Interestingly, when symptom measures were analyzed as time-varying covariates, neither anxiety nor depression showed substantive change over 12 months. Yet, within-person variation in dyspnea and energy displayed strong associations with anxiety: increasing breathlessness (β = 0.16; *P* < .001) and declining energy (β = 0.03; *P* < .001) corresponded to higher anxiety, whereas cough severity remained unrelated (e-Table 2). Depression scores followed a similar pattern. Age and cough severity contributed little, whereas greater dyspnea (β = 0.12; *P* < .001) and lower energy (β = 0.06; *P* < .001) were the principal correlates of worsening depression (e-Table 2). These relationships were consistent with the Spearman correlations linking changes in HADS scores with longitudinal shifts in PROs. Worsening breathlessness showed a moderate correlation with increased anxiety and depression scores (HADS-A: Spearman correlation coefficient *r* = 0.447; *P* < .001; HADS-D: *r* = 0.499; *P* < .001). Poorer energy levels were moderately associated with worsening depression (*r* = 0.407; *P* < .001), but only weakly correlated with worsening anxiety (*r* = 0.298; *P* < .001). Worsening cough weakly correlated with changes in HADS-A and HADS-D scores (Table 4). Changes in FVC % predicted showed no significant correlation with changes in HADS-A and HADS-D (Table 4).

**Table 4:** Spearman Correlations Between Changes in HADS Scores and Longitudinal Change in Lung Function and Patient-Reported Outcomes

Outcome	Δ HADS-Anxiety Score		Δ HADS-Depression Score	
	*r*	*P* Value	*r*	*P* Value
Δ FVC % predicted	−0.040	.660	−0.152	.090
Δ Cough VAS	0.210	.008	0.292	< .001
Δ LCQ	−0.200	.012	−0.147	.068
Δ Dyspnea-12	0.447	< .001	0.499	< .001
Δ L-IPF energy	0.298	< .001	0.407	< .001

HADS = Hospital Anxiety and Depression Scale; LCQ = Leicester Cough Questionnaire; L-IPF = Living with Idiopathic Pulmonary Fibrosis; VAS = visual analog scale.

**Figure 2:**
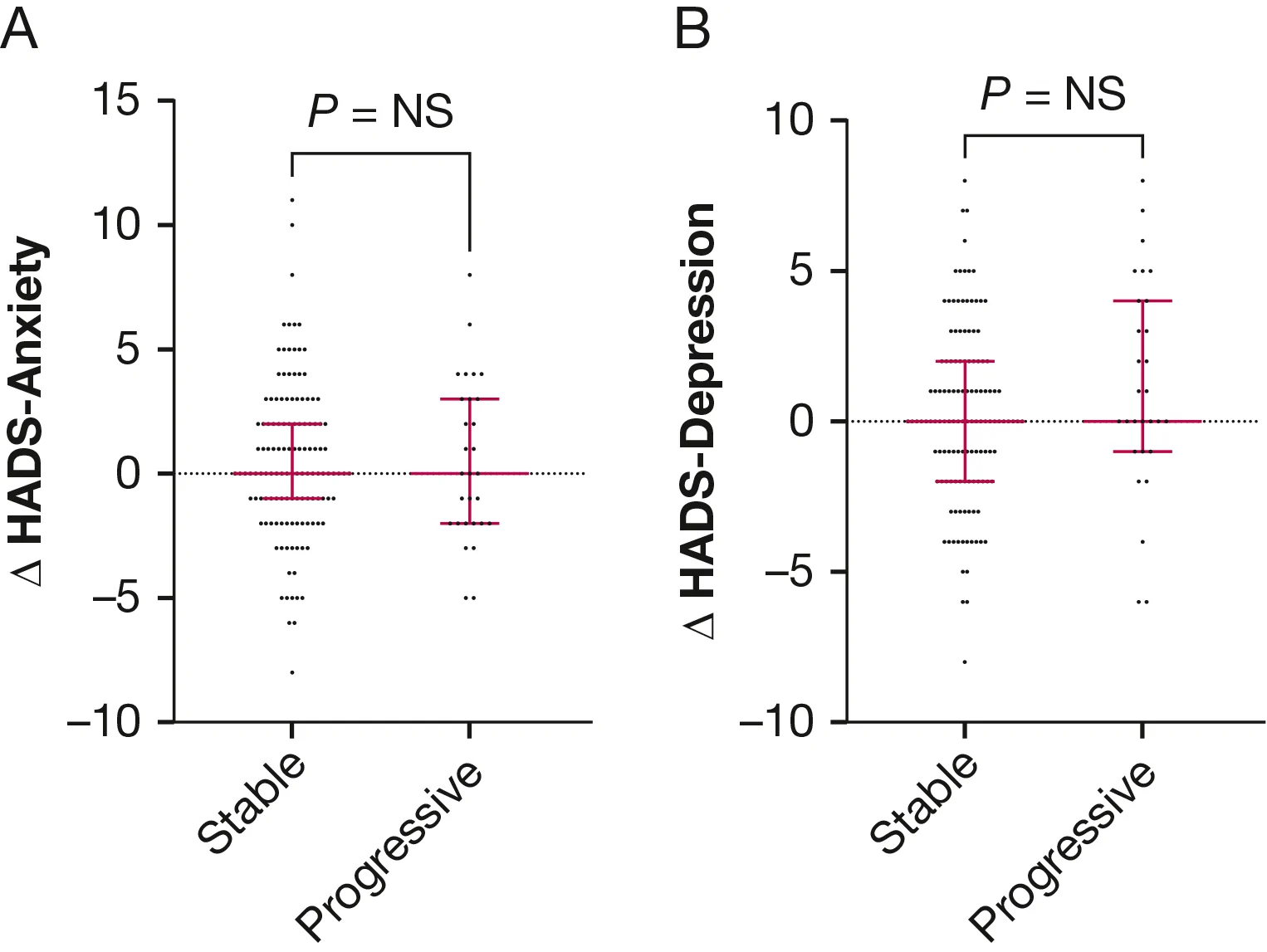
A, B, Scatterplot comparisons of 12-mo change (Δ) in (A) HADS anxiety and (B) HADS depression split into stable and progressive groups. Data are presented as medians and interquartile ranges. Progression was defined as a relative decline in % predicted FVC ≥ 10% or death within 12 mo. HADS = Hospital Anxiety and Depression Scale; NS = not significant.

No significant difference was found in HADS-A and HADS-D score change between patients with stable vs progressive disease (Fig 2).

## Discussion

Our analysis shows that anxiety and depression are common in patients with IPF. In this cohort of 269 patients with IPF, at baseline, 27.5% had anxiety and 28.3% had depression.

Our 12-month study suggests that anxiety and depression remain relatively stable throughout the disease course. Despite the statistically significant increase in HADS-A, this change is unlikely to be of clinical relevance given that previous studies report values for minimal clinically important differences that range from 2 (HADS-A) to 2.4 (HADS) in patients with IPF.^21^^,^^22^ In patients where there was a change in HADS-A, baseline factors consisting of a younger age, higher Dyspnea-12 scores, and poorer L-IPF energy levels were significant predictors of increased anxiety over time. Although the adjusted model highlighted significant associations of change in HADS-D over time with Dyspnea-12 and L-IPF energy scores, the overall model for depression was not statistically significant; therefore, these findings should be interpreted with caution.

No correlations were observed between changes in HADS scores and lung function. Weak to moderate correlations were identified between changes in HADS scores and longitudinal changes in PROs. Increasing breathlessness showed a moderate correlation with worsening anxiety and depression, whereas poorer energy levels demonstrated a moderate association with worsening depression. These patterns were supported by the mixed-effects models, in which dyspnea and energy, when treated as time-varying covariates, remained strongly linked to both anxiety and depression. In contrast, worsening cough severity had weak correlations with changes in HADS-A and HADS-D scores. Our analysis employed 2 complementary approaches. Baseline predictors were used to examine stable between-subject differences (ie, whether patients with higher baseline dyspnea or lower energy had worse anxiety/depression throughout), while time-varying covariates captured within-person changes (ie, whether an individual's worsening dyspnea or declining energy corresponded to their own increase in anxiety/depression). Both approaches demonstrated that dyspnea and energy were the key factors associated with psychological distress, reinforcing the robustness of these findings.

Our findings are in keeping with existing literature, where estimates for the prevalence of these conditions range from 21% to 60% for anxiety and 14% to 49% for depression in patients with ILD.^2^ In a large systematic review and meta-analysis conducted in an older adult population (> 60 years of age), the prevalence of anxiety was estimated to be 16.5% (95% CI, 11.1%-22.8%) and depression was estimated to be 19.2% (95% CI, 13.0%-27.5%).^23^ In comparison with this healthy population (without IPF), anxiety and depression were notably higher in our cohort of patients with IPF, who were of a similar age (average age, 73.5 years). This comparison highlights the effects of IPF on mental health.

In patients with COPD, previous studies have reported the prevalence of clinical anxiety in outpatients to range from 13% to 46%,^24^ whereas the prevalence of depression has been estimated at 26%.^25^ These findings are similar to those reported for patients with IPF, indicating the effect that chronic respiratory diseases can have on patient mental health.

Overall, our results indicate that dyspnea and low energy levels may have the greatest impact on anxiety and depression in patients with IPF, but that there is no relationship to disease severity as measured by lung function. This suggests that the worsening of these burdensome symptoms may have a greater impact on mood disorders than changes in lung function, a finding that has also been reported in an Australian IPF registry cohort.^5^ Interestingly, similar findings have been reported in patients with COPD. In a large cohort study that looked at the prevalence of depression in individuals with COPD, measures of QoL and symptoms were shown to be stronger determinants of depression than lung function.^25^ It is also possible that psychological factors, including anxiety and depression, may modulate patients’ perceptions of dyspnea and energy levels, rather than purely resulting from them.^26^^,^^27^ Further controlled interventional studies are warranted to investigate the directionality and potential causal nature of these associations.

Several other studies have reported the link between dyspnea and mood disorder in ILD. In a mixed cohort of 124 patients with ILD (48 with IPF), worse dyspnea (in terms of higher modified Medical Research Council dyspnea score) was an independent predictor of anxiety and depression.^14^ In a cross-sectional study of 63 patients with ILD from Brazil, the extent of dyspnea was shown to have a major impact on the physical and mental health-related QoL of patients.^28^ In a prospective cohort study of 52 individuals with ILD, depressive symptoms were strongly and independently correlated with dyspnea.^15^ In addition, the authors reported a strong link between pain and sleep quality.^15^ In contrast to our study and others that we have described, an association between depression and FVC was evident.^15^

This study has its limitations. Of the 269 participants, 112 (41.6%) did not have repeat HADS assessments at 12 months, including 47 deaths. Death represents an unavoidable form of missing data, as psychological outcomes cannot be assessed. Among those who died, 25.5% had baseline anxiety and 36.0% had baseline depression, suggesting a potential link between mental health and mortality; however psychological trajectories beyond baseline cannot be inferred. Our statistical approach accommodates for the missing data from the remaining 65 participants who were lost to follow-up for other reasons.

Anxiety and depression can be assessed using the HADS scale, a validated screening tool designed to identify suggestive and probable cases of anxiety and depression rather than providing a definitive clinical diagnosis.^29^ Although HADS-A and HADS-D are widely used and well-validated screening tools for anxiety and depression in chronic disease populations, they do not provide formal psychiatric diagnoses and have not been specifically validated in patients with IPF.^2^^,^^20^ The absence of follow-up data beyond 12 months also limits the ability to draw conclusions about the sustained trajectory of mood disorders over time. Although mental health status did not correlate with FVC decline in our study, both FVC and Dlco have previously been shown to correlate with health-related QoL outcomes in patients with ILD.^30^^,^^31^ These differences may reflect differences in study design, sample sizes, screening tools, and recruitment settings.

This cohort of 269 patients contributes meaningfully to the understanding of the psychological burden in IPF and is, to our knowledge, among the largest to assess mood disorders at the time of diagnosis. Recruiting patients at the point of diagnosis is a notable strength of the study, providing novel insight into the early psychological profile of individuals with newly diagnosed IPF.

## Interpretation

Anxiety and depression are prevalent in patients who are newly diagnosed with IPF and significantly associated with breathlessness and energy levels. Younger age was also an independent factor for greater anxiety. Our findings highlight the need for research into targeted symptom management, besides psychological support and disease-modifying strategies, to improve mental health and QoL of patients with IPF. Anxiety and depressive symptoms in patients with IPF should be regularly assessed, with appropriate support provided. Integrating mental health professionals into the multidisciplinary care team is essential because their understanding of IPF and its progression allows them to tailor treatments to the specific challenges faced by these patients, improving care and outcomes.

## Funding/Support

The work was supported by the 10.13039/501100000272National Institute for Health and Care Research through the 10.13039/501100013342Imperial Biomedical Research Centre, the NIHR Imperial Clinical Research Facility, and the Clinical Research Facility at Guy’s and St Thomas’ NHS Foundation Trust. P. L. M. is supported by the Asthma + Lung UK Chair award.

## Financial/Nonfinancial Disclosures

The authors have reported to *CHEST* the following: K. M. reports grants from Boehringer Ingelheim, AstraZeneca-Daiichi Sankyo, The Netherlands Organisation for Health Research and Development, and the Dutch Lung Foundation; speaker fees from Boehringer Ingelheim, AstraZeneca-Daiichi Sankyo, and GSK; and support for attending meetings from Boehringer Ingelheim. All grants and fees were paid to her institution. M. S. W. has received grants from AstraZeneca-Daiichi, Boehringer Ingelheim, Hoffmann-La Roche, The Netherlands Organisation for Health Research and Development, the Dutch Lung Foundation, and the Dutch Pulmonary Society; consulting fees from Agomab, Amgen, AstraZeneca, Avalyn, Boehringer Ingelheim, Galapagos, Bristol Myers Squibb, Calluna, Chiesi, Galapagos, Galecto, GSK, NeRRe Therapeutics, Horizon Therapeutics, PureTech Health, Kinevant Sciences, Molecure, CSL Behring, and Vicore; speaker fees from Boehringer Ingelheim, Galapagos, Hoffmann-La Roche, Novartis, and CSL Behring; and support for attending meetings from Boehringer Ingelheim, Galapagos, and Hoffmann-La Roche. All grants and fees were paid to her institution. C. S. and G. W. are employees of Boehringer Ingelheim International GmbH. T. M. M. has received speaker fees from Boehringer Ingelheim and Roche/Genentech and worked as a paid consultant for Boehringer Ingelheim, Roche/Genentech, AstraZeneca, Bayer, Blade Therapeutics, Bristol Myers Squibb, Galapagos, Galecto, GSK, IQVIA, Pliant Therapeutics, Respivant, Theravance, and Veracyte. P. L. M. reports consultancy fees from AstraZeneca, Boehringer Ingelheim, Endeavor, Hoffmann-La Roche, Qureight, Redx, Trevi, and Vicore; speaker fees from Boehringer Ingelheim and Hoffmann-La Roche; stock options from Qureight; and grant funding (paid to his institution) from Action for Pulmonary Fibrosis, Asthma + Lung UK, AstraZeneca, and GSK. None declared (Z. W.).

## Supplementary Data

e-Online Data
