# Supplementary material for: The Impact of Disease Severity and Symptoms on Anxiety and Depression in Individuals With Idiopathic Pulmonary Fibrosis
Source: Chest. 2026 Mar 30;170(2):467–76. doi: 10.1016/j.chest.2026.03.016 (PMC13494251; doi:10.1016/j.chest.2026.03.016)
Supplement: Supplementary file 1 [file mmc1.docx]

# The impact of disease severity and symptoms on anxiety and depression in individuals with idiopathic pulmonary fibrosis: Supplemental Material

Zhe Wu,^1^ Karen Moor,^2^ Marlies S. Wijsenbeek,^2^ Christina Schlecker,^3^ Gerrit Weimann,^3^ Toby M. Maher^1,4^, Philip L Molyneaux^1^

^1^National Heart and Lung Institute, Imperial College London, London UK; ^2^Department of Respiratory Medicine, Erasmus Medical Center, Rotterdam, the Netherlands; ^3^Boehringer Ingelheim International GmbH, Ingelheim am Rhein, Germany; ^4^Division of Pulmonary, Critical Care and Sleep Medicine, University of Southern California, Los Angeles, CA, USA.

## e-Appendix 1. Adjusted Linear Mixed-Effects Analysis of Predictors of HADS-Anxiety and HADS-Depression

For HADS-Anxiety, we adjusted *P* values across all fixed effects in the linear mixed effects model (time, age, baseline cough severity, baseline Dyspnea-12, and baseline energy). After Holm-Bonferroni correction, the effect of time remained non-significant (β=0.51, SE=0.26; adjusted *p*=0.11), as did baseline cough severity (β=−0.001, SE=0.01; adjusted *p*=0.92). In contrast, younger age remained significantly associated with lower anxiety scores (β=−0.13, SE=0.04; adjusted *p*=0.014), while higher baseline Dyspnea-12 (β=0.10, SE=0.04; adjusted *p*=0.035) and lower baseline energy (β=0.04, SE=0.02; adjusted *p*=0.023) remained significantly associated with higher anxiety levels after correction. These results indicate that the same covariates remained significant after controlling for multiple testing.

With HADS-Depression, following adjustment, baseline Dyspnea-12 remained significantly associated with greater depressive symptoms (β=0.16, SE=0.05; adjusted *p*=0.022), while baseline energy showed a borderline association (β=−0.045, SE=0.019; adjusted *p*=0.078). The effects of time (β=0.03, SE=0.26; adjusted *p*=1.00), age (β=0.003, SE=0.056; adjusted *p*=1.00), and baseline cough severity (β=0.004, SE=0.017; adjusted *p*=1.00) were non-significant.

**e-Table 1. Clinical Variables Associated with Anxiety (HADS-A) and Depression (HADS-D) at Baseline (Univariable Analyses)**

|  | **HADS-A** | | |  | **HADS-D** |  |
| --- | --- | --- | --- | --- | --- | --- |
| **Variables** | **β** | **95% CI (β)** | ***P* Value** | **β** | **95% CI (β)** | ***P* Value** |
| Age | -0.107 | -0.166 to -0.047 | **<0.001** | -0.022 | -0.085 to 0.042 | 0.504 |
| Male sex | -0.989 | -2.120 to 0.142 | 0.086 | -0.017 | -1.203 to 1.169 | 0.978 |
| BMI | 0.059 | -0.037 to -0.155 | 0.229 | 0.131 | 0.031 to 0.230 | **0.010** |
| Smoking history | 1.241 | 0.314 to 2.168 | **0.009** | 1.829 | 0.872 to 2.786 | **<0.001** |
| Oxygen therapy | 1.600 | 0.217 to 2.983 | **0.024** | 1.893 | 0.455 to 3.331 | **0.010** |
| ACEi therapy | -0.359 | -1.443 to 0.725 | 0.515 | 0.813 | -0.315 to 1.941 | 0.157 |
| FVC% predicted | -0.007 | -0.037 to 0.024 | 0.664 | -0.040 | -0.072 to -0.009 | **0.012** |
| DL_CO_% predicted | -0.010 | -0.043 to 0.022 | 0.528 | -0.042 | -0.076 to -0.009 | **0.014** |
| Dyspnea-12 | 0.196 | 0.151 to 0.240 | **<0.001** | 0.228 | 0.183 to 0.273 | **<0.001** |
| Cough VAS | 0.030 | 0.011 to 0.048 | **<0.001** | 0.031 | 0.012 to 0.050 | **0.001** |
| LCQ | -0.430 | -0.587 to -0.273 | **<0.001** | -0.297 | -0.465 to -0.130 | **<0.001** |
| L-IPF impacts | 0.060 | 0.042 to 0.078 | **<0.001** | 0.093 | 0.077 to 0.109 | **<0.001** |
| L-IPF cough | 0.047 | 0.027 to 0.068 | **<0.001** | 0.045 | 0.023 to 0.066 | **<0.001** |
| L-IPF dyspnea | 0.058 | 0.032 to 0.083 | **<0.001** | 0.098 | 0.074 to 0.123 | **<0.001** |
| L-IPF energy | 0.071 | 0.050 to 0.092 | **<0.001** | 0.112 | 0.093 to 0.130 | **<0.001** |

Data are presented with unstandardized (β) correlation coefficients with 95% CIs for associations between factors and HADS scores.

ACEi, angiotensin-converting enzyme inhibitor; BMI, body mass index; CI, confidence interval; DL_CO_, diffusing capacity of the lungs for carbon monoxide; FVC, forced vital capacity; HADS, Hospital Anxiety and Depression Scale; LCQ, Leicester Cough Questionnaire; L-IPF, Living with Idiopathic Pulmonary Fibrosis; VAS, visual analog scale.

## e-Table 2. Linear Mixed-Effects Model Examining 12-Month Change in HADS Scores.

| **Model** | **REML Criterion** | **Random Intercept Variance** | **Residual Variance** | **Time Effect Estimate** | **Time Effect Standard Error** | **Time Effect**  ***P* Value** | **Covariate** | | |
| --- | --- | --- | --- | --- | --- | --- | --- | --- | --- |
|  |  |  |  |  |  |  |  | **β Coefficient** | ***P* Value** |
| **HADS-Anxiety** | | | | | | | | | |
| Unadjusted | 2252.0 | 9.418 | 4.857 | 0.482 | 0.240 | **0.044** |  | | - |
| Adjusted (for baseline covariate values) | 1433.8 | 7.771 | 4.793 | 0.506 | 0.264 | 0.057 | Age  Cough VAS  Dyspnea-12  L-IPF energy | -0.125  -0.001  0.104  0.044 | **0.003**  0.925  **0.011**  **0.006** |
| Adjusted (with time varying covariates) | 1371.2 | 8.006 | 3.534 | 0.442 | 0.235 | 0.062 | Age  Cough VAS  Dyspnea-12  L-IPF energy | -0.117  0.004  0.162  0.034 | **0.003**  0.633  **< 0.001**  **< 0.001** |
| **HADS-Depression** | | | | | | | | | |
| Unadjusted | 2373.4 | 19.444 | 4.891 | -0.049 | 0.242 | 0.838 |  | | - |
| Adjusted (for baseline covariate values) | 1681.7 | 19.295 | 4.629 | 0.027 | 0.258 | 0.918 | Age  Cough VAS  Dyspnea-12  L-IPF energy | -0.002  0.004  0.156  -0.045 | 0.962  0.791  **0.004**  **0.016** |
| Adjusted (with time varying covariates) | 1321.4 | 4.809 | 3.203 | 0.000 | 0.223 | 1.000 | Age  Cough VAS  Dyspnea-12  L-IPF energy | 0.022  0.005  0.122  0.063 | 0.499  0.455  **< 0.001**  **< 0.001** |

The independent variable ‘Time’ represents data point comparisons between baseline and 12 months.

HADS, Hospital Anxiety and Depression Scale; L-IPF, Living with Idiopathic Pulmonary Fibrosis; REML, restricted maximum likelihood; VAS, visual analog scale.
